# Magnetic resonance imaging-based lower limb muscle evaluation in Charcot-Marie-Tooth disease type 1A patients and its correlation with clinical data

**DOI:** 10.1038/s41598-022-21112-8

**Published:** 2022-10-05

**Authors:** Yeo Jin Kim, Hyun Su Kim, Ji Hyun Lee, Young Cheol Yoon, Byung-Ok Choi

**Affiliations:** 1 Department of Radiology, Veterans Health Service Medical Center, Seoul, 05368 South Korea; 2 Department of Radiology, Samsung Medical Center, Sungkyunkwan University School of Medicine, 81 Irwon-ro, Gangnam-gu, Seoul, 06351 South Korea; 3 Department of Neurology, Samsung Medical Center, Sungkyunkwan University School of Medicine, Seoul, 06351 South Korea

**Keywords:** Neuromuscular disease, Biomarkers, Predictive markers

## Abstract

We aimed to derive comprehensive MRI parameters that reflect intramuscular fat infiltration severity for designated lower extremity levels, based on semiquantitative analyses in Charcot-Marie-Tooth disease type 1A (CMT1A) patients. We reviewed lower extremity MRIs of 116 CMT1A patients. Intramuscular fat infiltration grading using the Mercuri scale was performed for the non-dominant lower extremity at three levels (proximal, mid, and distal) for the thigh and at two levels (proximal and distal) for the lower leg. Based on MRI results, the following parameters were calculated for each level and for entire muscles: fat infiltration proportion (FIP), significant fat infiltration proportion (SigFIP), and severe fat infiltration proportion (SevFIP). The relationships between the MRI parameters and clinical data were evaluated using Spearman’s correlation analysis. FIP, SigFIP, and SevFIP measured for entire muscles significantly correlated with Charcot-Marie-Tooth Neuropathy Score (*p* < 0.001), functional disability scale (*p* < 0.001), 10-m walk test time (*p* = 0.0003, 0.0010, and 0.0011), and disease duration (*p* < 0.001). Similar correlations were demonstrated for FIP, SigFIP, and SevFIP acquired from the lower leg. Our MRI parameters obtained through semiquantitative analyses of muscles significantly correlated with clinical parameters in CMT1A patients, suggesting their potential applicability as imaging markers for clinical severity.

## Introduction

Charcot-Marie-Tooth disease (CMT), the most common hereditary neuromuscular disorder, comprises a group of genetically and clinically heterogeneous disorders characterized by symmetric distal muscle wasting, muscle weakness, and sensory loss^1,2^. The most common type of CMT, CMT type 1A (CMT1A), results from a duplication of the peripheral myelin protein 22 (PMP22) gene on chromosome 17^1^. There is a substantial variability in the clinical course of patients with CMT1A, and a significant age-dependent increase in neurologic deficits can occur^2,3^; hence, these patients should be examined carefully. Although there is currently no cure for the disease, several recent clinical and preclinical studies using therapeutic agents have shown encouraging results^4–6^. In this regard, it is necessary to develop and establish an objective and reproducible evaluation method for patients with CMT1A, including the evaluation of treatment response.

The clinical assessment of patients with CMT1A has traditionally relied on physical examination and electrophysiological studies^1^. Owing to the demand for additional clinical information and objective evaluation tools, various diagnostic imaging studies have been increasingly employed in clinical practice^3,7,8^. Several studies have targeted a number of anatomical structures using different imaging techniques in patients with CMT1A and reported promising results relating to the use of potential imaging biomarkers for these patients^9–13^. An MRI evaluation of fat infiltration in extremity muscles has been the most widely used of these techniques not only for patients with CMT1A, but also for those with other neuromuscular disorders, as extremity muscle fat infiltration is the central pathophysiological mechanism directly linked to the clinical manifestations of CMT1A^7,9,14–17^. The evaluation of intramuscular fat infiltration using MRI can be accomplished via a semiquantitative or quantitative approach. The semiquantitative approach is performed by evaluating each muscle using a classification system such as the Mercuri or Goutallier scale, which grades a muscle based on the relative amount of fat tissue present within the muscle^7,16^. The quantitative approach is performed by obtaining an additional imaging sequence containing a fat fraction map that enables a direct quantitative measurement of the fat percentage of each muscle^7,9^. Both approaches focus on the evaluation of individual muscles, rather than providing comprehensive information on the overall severity of fat infiltration at the designated level. To our knowledge, few studies have evaluated imaging parameters that comprehensively reflect the degree of muscular fat infiltration based on semiquantitative MRI in patients with CMT.

In this study, we aimed to derive comprehensive MRI parameters that reflect the severity of intramuscular fat infiltration for designated levels of the lower extremity, based on semiquantitative analyses using the Mercuri scale in patients with CMT1A. We assessed the potential value of these MRI parameters as imaging biomarkers by correlating them with clinical parameters. In addition, we evaluated the degree and pattern of intramuscular fat infiltration at multiple levels of the lower extremity muscles.

## Methods

### Study participants

The institutional review board of our institution (Samsung Medical Center Institutional Review Board, file no. 2020–05-115–003) approved this retrospective study and waived the requirement for informed consent due to the retrospective nature of the study. The study was conducted in accordance with the declaration of Helsinki. Initially, we enrolled 223 patients diagnosed with CMT1A, who were confirmed to have PMP22 duplication by genetic analysis at our neurology department, and who underwent lower extremity MRI between September 2013 and March 2020. Although significant intrafamilial variability in clinical manifestations has been reported in patients with CMT1A^18^, we chose the oldest member of each family for analysis, considering the potential similarity of muscular fat infiltration among family members (thereby excluding 65 subjects). We additionally excluded 42 patients who had missing clinical assessment data. Furthermore, we sought to exclude patients with previous lower extremity surgery or suboptimal MRI quality; however, no such patients were present.

### MRI acquisition

Lower extremity MRI scans were obtained using a 1.5-T or 3.0-T MRI system (Avanto or Skyra, Siemens Healthcare, Frankfurt, Germany; Ingenia, Philips Healthcare, Best, Netherlands) with a 16-channel anterior coil and a posterior built-in coil. Axial T1-weighted images encompassing the pelvic girdle, bilateral thighs, and lower legs were obtained at different levels from the anterior inferior iliac spine through the distal tibia. Imaging parameters were as follows: repetition time, 613.7 ms; echo time, 16.7 ms; flip angle, 90 $^\circ$; number of signal averaged, 1; reconstructed voxel size, 0.68 mm; matrix size, 320 $\times$ 320; field of view, 350 $\times$ 350 mm; section thickness, 2 mm; and gap, 1 mm. Imaging times were 162 and 180 s for the thigh and lower leg, respectively.

### Image analysis

Two radiologists (Y.C.Y and H.S.K with 18 and 9 years of experience in musculoskeletal radiology, respectively) performed a consensus-based analysis of the thigh and lower leg muscles using T1-weighted images. Intramuscular fat infiltration grading was performed using the Mercuri scale^8,19^ as follows: stage 0, normal appearance; stage 1, scattered small areas of increased signal; stage 2a, numerous discrete areas of increased signal comprising less than 30% of the muscle; stage 2b, numerous discrete areas of increased signal comprising 30–60% of the muscle; stage 3, washed-out appearance due to confluent areas of increased intensity with muscle still present at the periphery; and stage 4, end-stage appearance, muscle entirely replaced by areas of increased signal.

**Figure 1:**
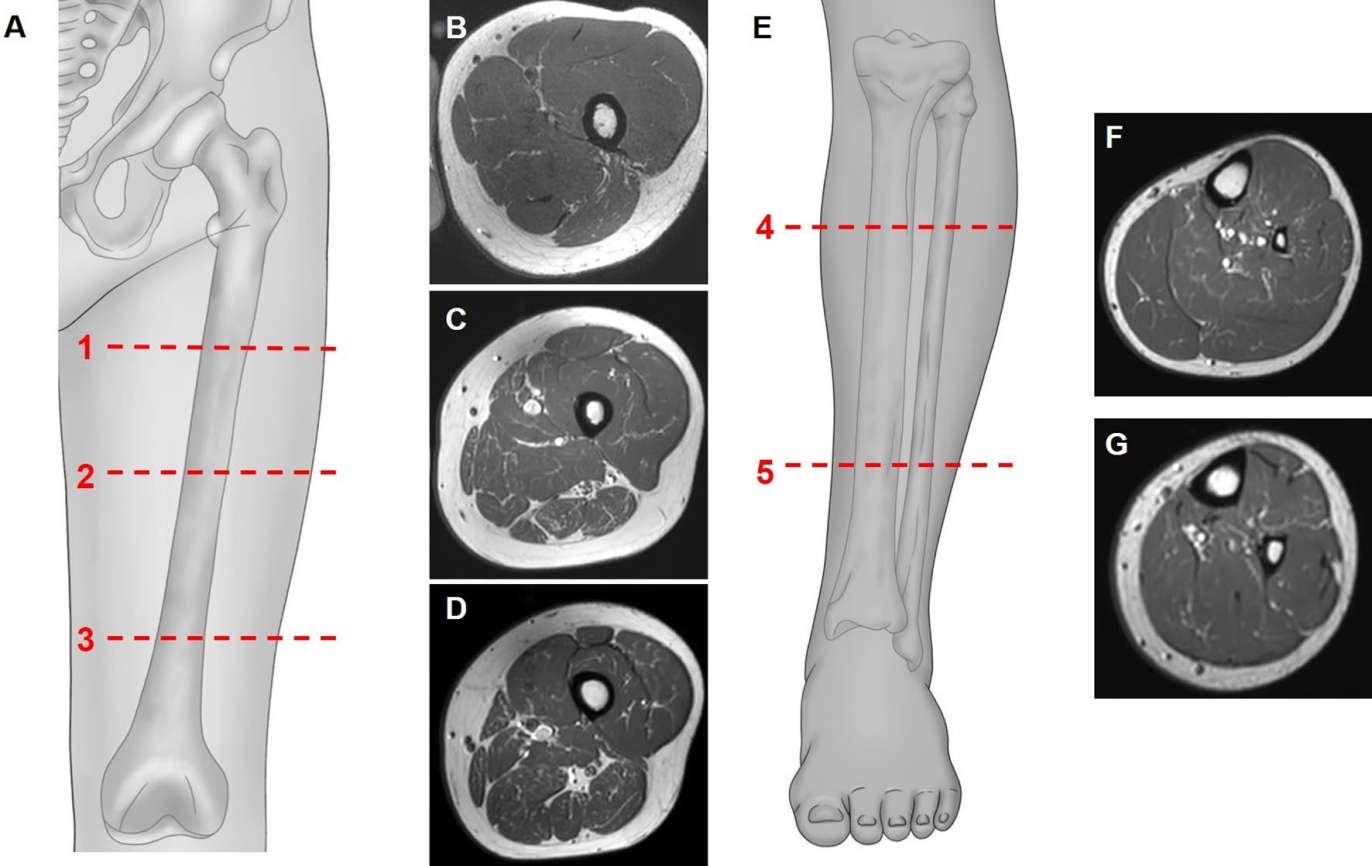
Illustrations (**A** and **E**) and axial (**B**, **C**, **D**, **F**, and **G**) T1-weighted images of the left lower extremity showing the levels where analyses were performed. A and E depict the levels where analyses were performed. Axial T1-weighted images of the thigh (**B**, **C**, and **D**) and lower leg (**F** and **G**) where gradings of the intramuscular fat infiltration using the Mercuri scale were performed: gluteus maximus tendon insertion (**B**, level 1, proximal thigh); just inferior to the gluteus maximus inferior margin (**C,** level 2, mid thigh); just inferior to the adductor longus inferior margin (**D**, level 3, distal thigh); just inferior to the popliteus inferior margin (**F**, level 4, proximal lower leg); and the uppermost part of the gastrocnemius tendon (**G**, level 5, distal lower leg). Illustrations (**A** and **E**) were made using Adobe Photoshop software version 21.0 ( Adobe Systems, San Jose, CA).

Evaluations were performed for the non-dominant lower extremity at three levels (proximal, mid, and distal) for the thigh muscles and at two levels (proximal and distal) for the lower leg muscles. Levels were determined based on anatomical landmarks on axial T1-weighted images: gluteus maximus tendon insertion (proximal thigh, level 1); just inferior to the inferior margin of the gluteus maximus, where the muscle is no longer visualized (mid-thigh, level 2); just inferior to the inferior margin of the adductor longus, where the muscle is no longer visualized (distal thigh, level 3); just inferior to the inferior margin of the popliteus, where the muscle is no longer visualized (proximal lower leg, level 4); and the uppermost part of the gastrocnemius tendon, where the gastrocnemius muscle is no longer visualized (distal lower leg, level 5) (Fig. 1).

The following muscles were evaluated at each level: sartorius, rectus femoris, vastus lateralis, vastus medialis, vastus intermedius, adductor longus, adductor brevis, adductor magnus, gracilis, and semitendinosus (level 1, 10 muscles); sartorius, rectus femoris, vastus lateralis, vastus medialis, vastus intermedius, adductor longus, adductor brevis, adductor magnus, gracilis, semitendinosus, semimembranosus, and biceps femoris (level 2, 12 muscles); sartorius, rectus femoris, vastus lateralis, vastus medialis, vastus intermedius, gracilis, semitendinosus, semimembranosus, and biceps femoris (level 3, 9 muscles); tibialis anterior, extensor digitorum longus, peroneus longus, gastrocnemius medialis, gastrocnemius lateralis, soleus, and tibialis posterior (level 4, 7 muscles); tibialis anterior, extensor digitorum longus, extensor hallucis longus, peroneus longus, soleus, tibialis posterior, flexor digitorum longus, and flexor hallucis longus (level 5, 8 muscles).

**Figure 2:**
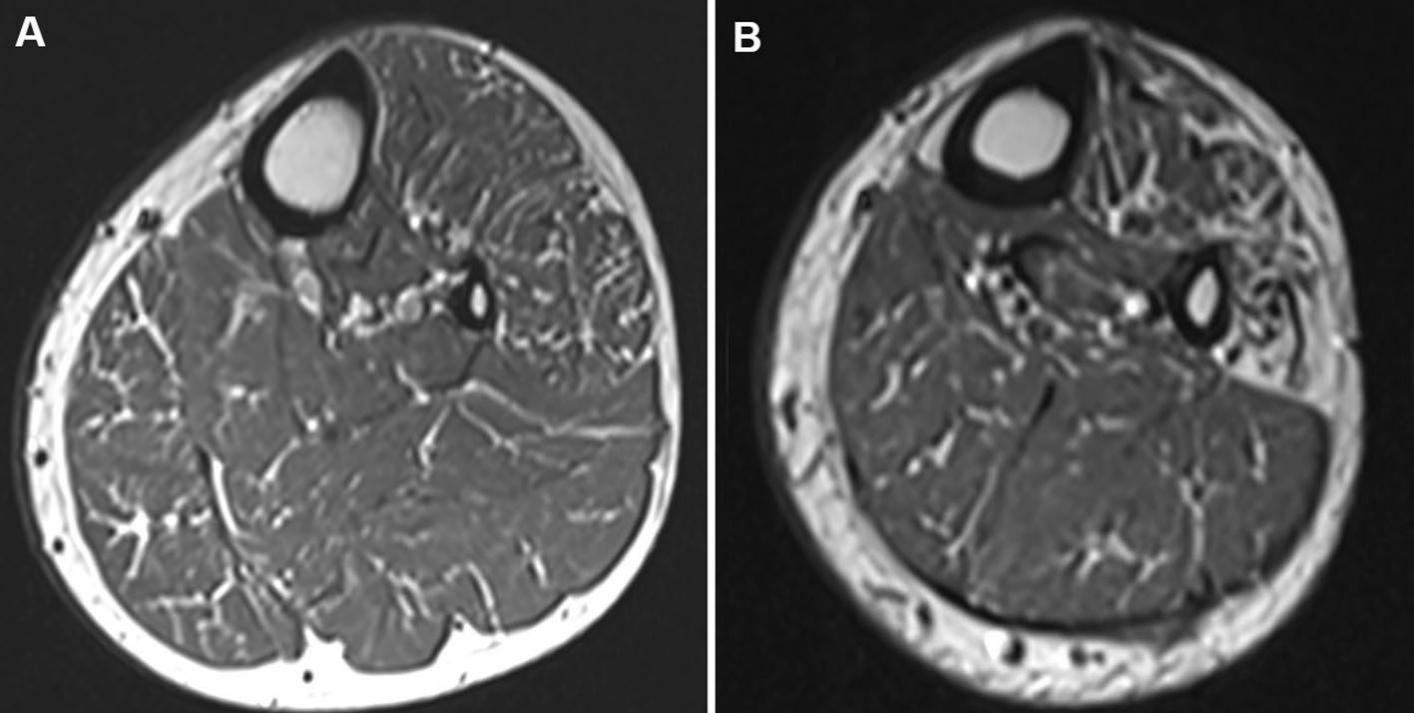
Axial T1-weighted images of the proximal (**A**, level 4) and distal (**B**, level 5) lower leg in a 30-year-old man with Charcot-Marie-Tooth disease type 1A. Intramuscular fat infiltration was graded using the Mercuri scale at proximal lower leg (**A**) as follows: tibialis anterior, 1; extensor digitorum longus, 1; peroneus longus, 2a; tibialis posterior, 1; soleus, 1; gastrocnemius medialis, 2a; and gastrocnemius lateralis, 1. Fat infiltration proportion (FIP), significant fat infiltration proportion (SigFIP), and severe fat infiltration proportion (SevFIP) were calculated using the following formulas: FIP = number of muscles with stage 1 or higher / number of all evaluated muscles x 100 (%); SigFIP = number of muscles with stage 2a or higher / number of all evaluated muscles x 100 (%); and SevFIP = number of muscles with stage 3 or higher / number of all evaluated muscles x 100 (%). Acquired parameters for this patient were: FIP = 100%, Sig FIP = 28.6%, and SevFIP = 0% at level 4. MRI obtained at the distal lower leg (**B**) shows preferential fat infiltration in the anterior and lateral compartment muscles.

Based on the MRI results, we defined the following quantitative parameters to measure the degree of muscular fat infiltration at each level (Fig. 2).

- Fat infiltration proportion (FIP) = number of muscles with stage 1 or higher/total number of evaluated muscles × 100 (%)
- Significant fat infiltration proportion (SigFIP) = number of muscles with stage 2a or higher/total number of evaluated muscles × 100 (%)
- Severe fat infiltration proportion (SevFIP) = number of muscles with stage 3 or higher/total number of evaluated muscles × 100 (%)

Each parameter was also assessed for the entire muscles (46 muscles) of the lower extremity (total FIP, SigFIP, and SevFIP).

### Clinical assessments

We assessed clinical data for patients with CMT1A, including age at onset, disease duration, CMT Neuropathy Score version 2 (CMTNSv2), 10 m walk test (10-MWT) time, and functional disability scale (FDS) score. The age at onset was determined by asking the patient when symptoms such as foot deformity, distal muscle weakness, and/or sensory changes initially appeared. The CMTNSv2 is a composite score consisting of neurological symptoms, clinical signs, and electrophysiological parameters. It ranges from 0 (no deficit) to 36 (maximum deficit)^20^. A nine-point FDS was used to determine disease severity in terms of the ability to walk and run as follows: 0 = normal; 1 = normal, but with cramps and fatigability; 2 = inability to run; 3 = walking difficult, but still possible unaided; 4 = able to walk with a cane; 5 = able to walk with crutches; 6 = able to walk with a walker; 7 = wheelchair-bound; and 8 = bedridden^18^. For the 10-MWT, which was performed to evaluate locomotor ability, patients were asked to walk a 10-m distance, and the time taken was measured in seconds.

### Statistical analysis

Relationships between MRI parameters (FIP, SigFIP, and SevFIP for each level and for entire extremity muscles) and clinical data (age at onset, disease duration, CMTNSv2, FDS score, and 10-MWT time) were evaluated using Spearman’s correlation analysis in patients with CMT1A. Descriptive statistics for MRI parameters, clinical variables, and demographic variables are presented as mean ± standard deviation, median [interquartile range], and range. Statistical analyses were performed using SAS version 9.4 (SAS Institute, Cary, NC, USA). A *p* value < 0.05 was considered statistically significant. Spearman’s correlation coefficients were interpreted as follows: *r* < 0.2, negligible correlation; 0.2 ≤ *r* < 0.4, weak correlation; 0.4 ≤ r < 0.6, moderate correlation; 0.6 ≤ r < 0.8, strong correlation; and *r* ≥ 0.8, very strong correlation^21^.

## Results

### Patient characteristics

Clinical and demographic data of patients with CMT1A are shown in Table 1. 116 patients with CMT1A (63 men, 53 women; age range, 8–78 years; mean age, 41.7 ± 17.1 years) were included in the study. The age at onset and disease duration ranged from 1 to 77 years (median, 16 [8–43] years) and 0 to 66 years (median, 10 [4–26] years), respectively. The CMTNSv2 and FDS score ranged from 5 to 32 (median, 16 [12.5–19.5]) and 0 to 7 (median, 2 [1–2.5]), respectively. The 10-MWT time ranged from 6.0 to 31.6 s (median, 10.8 [8.2–14.8] s). The right and left lower extremities were dominant in 113 and 3 patients, respectively.

**Table 1:** Clinical and demographic data of patients with Charcot-Marie-Tooth disease type 1A.

Parameters		
Sex (person)	Male	63 (54.3%)*
	Female	53 (45.7%)*
Age at exam (years)	Median (IQR)	44 (26-55)
Age at onset (years)	Median (IQR)	16 (8-43)
	≤10 years	40 (34.5%)*
	>10 years	76 (65.5%)*
Disease duration (years)	Median (IQR)	10 (4-26)
	≤10 years	62 (53.4%)*
	>10 years	54 (46.6%)*
CMTNSv2 (0-36)	Median (IQR)	16 (12.5-19.5)
	Mild (0-10)	16 (13.8%)*
	Moderate (11-20)	76 (65.5%)*
	Severe (21-36)	24 (20.7%)*
FDS score (0-8)	Median (IQR)	2 (1-2.5)
	Mild (0-2)	89 (76.7%)*
	Severe (3-8)	27 (23.3%)*
10-MWT time (sec)	Median (IQR)	10.8 (8.2-14.8)

*Data are expressed as the number of patients with percentages in parentheses.

*CMTNSv2* Charcot-Marie-Tooth Neuropathy Score version 2; *FDS* Functional Disability Scale; *10-WMT* 10-m walk test; *IQR* interquartile range.

### Imaging analyses results

**Figure 3:**
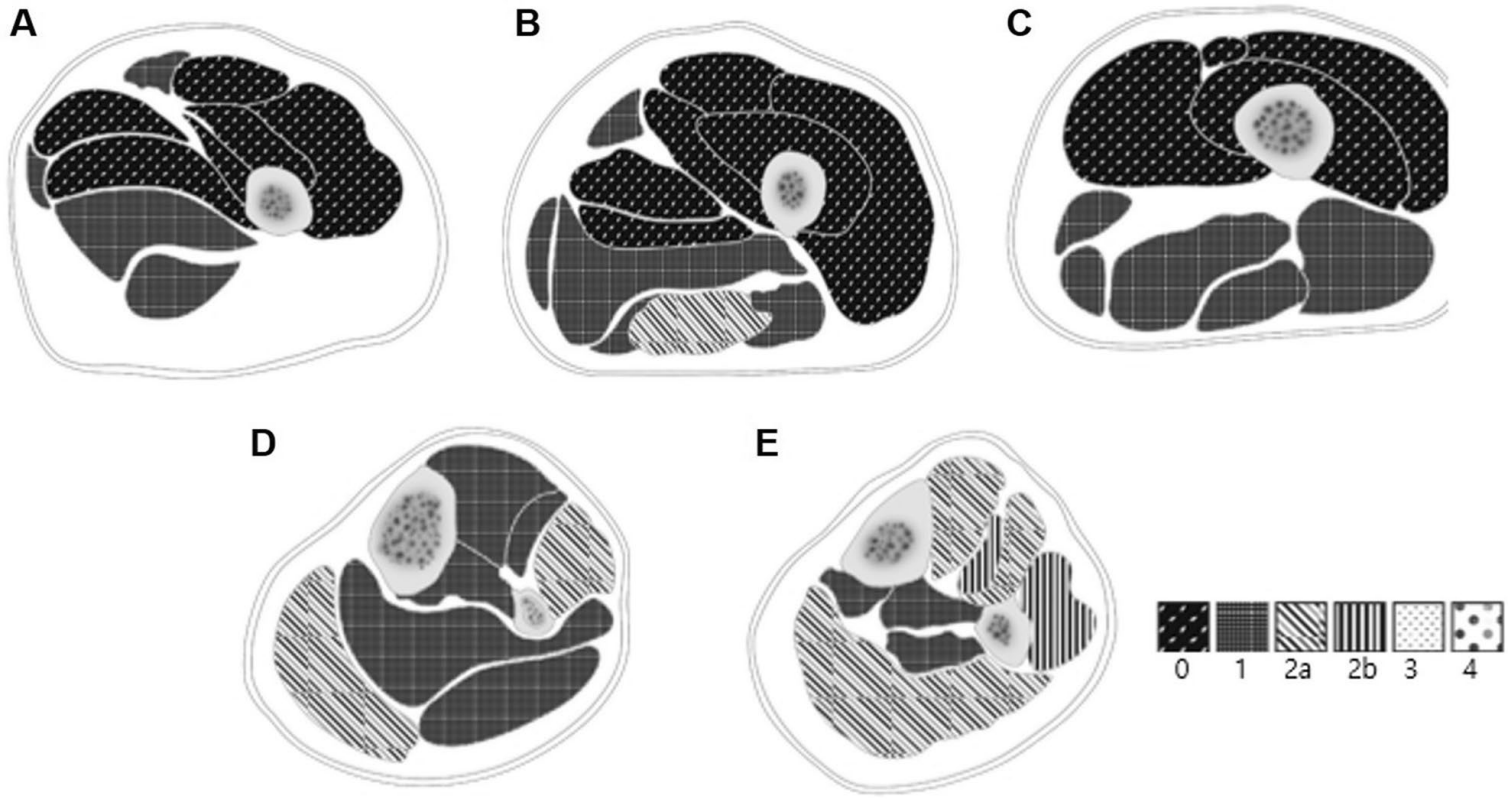
Schematic diagram depicting median Mercuri stages for individual muscles at levels 1–5 (**A**–**E**). The median Mercuri stages of evaluated muscles were highest (stage 2b) in the extensor hallucis longus and peroneus longus at level 5 (**E**). Other anterior and superficial posterior compartment muscles at level 5, as well as the peroneus longus and gastrocnemius medialis at level 4, demonstrated a median Mercuri stage of 2a (**D** and **E**). Among thigh muscles, the highest median Mercuri stage was found in the semitendinosus muscle at level 2 (stage 2a) (**B**). Illustrations were made using Adobe Photoshop software version 21.0 (Adobe Systems, San Jose, CA).

The results of the imaging analyses, including measured FIP, SigFIP, and SevFIP at each level, are summarized in Supplementary Tables S1–S3. Figure 3 shows a schematic diagram depicting the median Mercuri stage for individual muscles at each level. The median Mercuri stage of the evaluated muscles was highest in the extensor hallucis longus and peroneus longus at level 5 (stage 2b), thereby suggesting that 30–60% of the areas within the designated muscles were infiltrated with fat. Other anterior and superficial posterior compartment muscles at level 5, as well as the peroneus longus and gastrocnemius medialis at level 4, demonstrated a median stage of 2a, thereby suggesting that less than 30% of the areas within the muscles were infiltrated with fat. Among the thigh muscles, the highest median Mercuri stage was found in the semitendinosus muscle at level 2 (stage 2a).

### Correlation between MRI and clinical parameters

The results of the correlation analysis between the MRI and clinical parameters are summarized in Table 2. MRI parameters measured for entire muscles (total FIP, SigFIP, and SevFIP) were significantly correlated with CMTNSv2, FDS score, 10-MWT time, and disease duration. Similarly, we observed correlations between MRI parameters acquired from the lower leg (levels 4 and 5) and CMTNSv2, FDS score, 10-MWT time, and disease duration. Furthermore, MRI parameters acquired from the thigh level (level 1–3) were significantly correlated with clinical parameters, although these correlations were less consistent than those mentioned above. No significant correlation was found between age at onset and MRI parameters.

**Table 2:** Correlation analysis between MRI and clinical parameters in patients with Charcot-Marie-Tooth disease type 1A.

MRI parameters	Level	CMTNSv2		FDS score		10-MWT time		Disease duration		Age at onset	
		*r*	*p*	*r*	*p*	*r*	*p*	*r*	*p*	*r*	*p*
FIP	Total	0.3586	<.0001*	0.4267	<.0001*	0.3267	0.0003*	0.3084	0.0008*	0.0593	0.5271
	1	0.2731	0.003*	0.3129	0.0007*	0.2570	0.0054*	0.2845	0.0020*	0.0599	0.5229
	2	0.1692	0.0694	0.2409	0.0095*	0.1785	0.0553	0.1723	0.0643	0.0365	0.6974
	3	0.2180	0.0188*	0.2691	0.0036*	0.1814	0.0513	0.1106	0.2373	-0.0035	0.9704
	4	0.3761	<.0001*	0.4269	<.0001*	0.2650	0.0040*	0.3335	0.0003*	0.1695	0.0689
	5	0.2722	0.0031*	0.3716	<.0001*	0.2234	0.0159*	0.2489	0.0071*	0.1393	0.1358
SigFIP	Total	0.3490	0.0001*	0.4328	<.0001*	0.3008	0.0010*	0.4057	<.0001*	0.0515	0.5832
	1	0.1421	0.1281	0.1879	0.0443*	0.2059	0.0266*	0.2459	0.0078*	-0.0181	0.8473
	2	0.1527	0.1018	0.2187	0.0189*	0.1459	0.1182	0.3199	0.0005*	-0.0030	0.9744
	3	0.2102	0.0235*	0.2752	0.0029*	0.1613	0.0836	0.3315	0.0003*	-0.0289	0.7581
	4	0.3774	<.0001*	0.5292	<.0001*	0.2971	0.0012*	0.3767	<.0001*	0.0645	0.4918
	5	0.4352	<.0001*	0.5170	<.0001*	0.3091	0.0007*	0.3862	<.0001*	0.1002	0.2846
SevFIP	Total	0.3793	<.0001*	0.4522	<.0001*	0.3004	0.0011*	0.3561	<.0001*	0.0627	0.5036
	1	0.1605	0.0853	0.1514	0.1062	0.1295	0.1658	0.1491	0.1101	-0.1005	0.2831
	2	0.2004	0.0310*	0.2236	0.0163*	0.1997	0.0317*	0.1814	0.0513	-0.1427	0.1264
	3	0.1953	0.0357*	0.2127	0.0225*	0.1388	0.1372	0.1955	0.0354*	-0.1460	0.1178
	4	0.3917	<.0001*	0.4885	<.0001*	0.2531	0.0061*	0.3329	0.0003*	-0.0633	0.4999
	5	0.3257	0.0004*	0.4023	<.0001*	0.2484	0.0072*	0.3560	<.0001*	0.0986	0.2924

*Indicates statistical significance.

*r* Spearman’s correlation coefficient; *p* *p*-value; *FIP* fat infiltration proportion; *Sig FIP* significant fat infiltration proportion; *SevFIP* severe fat infiltration proportion; *CMTNSv2* Charcot-Marie-Tooth Neuropathy Score version 2; *FDS* Functional Disability Scale; *10-WMT* 10-m walk test.

All three MRI parameters acquired from the entire muscles and those acquired from the lower leg level (levels 4 and 5) showed moderate correlations with FDS score. In addition, SigFIP at level 5 and total SigFIP were moderately correlated with CMTNSv2 and disease duration, respectively. FDS score and SigFIP at level 4 had the strongest correlation (*r* = 0.5292), followed by FDS score and SigFIP at level 5 (*r* = 0.5170). Degree of other significant correlations between MRI parameters and clinical parameters were weak or negligible correlation.

## Discussion

In this study, we proposed MRI parameters that may comprehensively reflect intramuscular fat infiltration severity at designated levels; these parameters were obtained through semiquantitative analyses of lower extremity muscles using the Mercuri scale in patients with CMT1A. MRI parameters acquired from entire muscles and those acquired from the lower leg levels (levels 4 and 5) showed moderate positive correlations with clinical parameters; FDS score showed the strongest correlation with SigFIP at level 4.

Our proposed MRI parameters were calculated as proportions of muscles that showed fat infiltration among all individual muscles at designated levels. We devised three parameters—FIP, SigFIP, and SevFIP—to represent proportions of muscles showing fat infiltration (Mercuri stage 1 or higher), significant fat infiltration (Mercuri stage 2a or higher), and severe fat infiltration (Mercuri stage 3 or higher), respectively. We devised separate imaging parameters to verify if the proportion of muscles with significant (SigFIP) or severe fat infiltration (SevFIP) showed a more significant correlation with clinical parameters compared to the correlation between proportions of any individual muscles that display fat infiltration (FIP). All three parameters showed meaningful correlations with the clinical parameters, without significant differences among each other. In clinical practice, it may be challenging to determine whether mild intramuscular fat infiltration identified on MRI is related to the normal aging process or results from denervation in patients with neuromuscular disorders^22^. Our result does not seem to imply that only the proportion of muscles with substantial or severe fat infiltration possess more grave clinical significance. It may be necessary conduct a large-scale study to evaluate which MRI parameter is more strongly correlated with clinical parameters and to assess the potential efficacies of these MRI parameters as imaging biomarkers in patients with CMT1A.

Our results showed moderate positive correlations between MRI parameters and FDS score as well as weak positive correlations between MRI parameters and 10-MWT time. Both the FDS score and 10-MWT time are used to assess disease severity in terms of the ability to walk and run. Our results may suggest a potential applicability of MRI parameters in evaluating motor function in patients with CMT1A. In addition, our findings revealed that clinical parameters were more strongly correlated with MRI parameters obtained from the lower leg level than with those obtained from the thigh level. Distally predominant limb muscle wasting is a characteristic feature in patients with CMT1A^1,2^, and therefore our findings may provide scope for further studies to enhance the application of MRI analyses at multiple levels in the lower leg level than in the thigh level.

MRI parameters were correlated with CMTNSv2, which is a composite scoring system comprising several factors including clinical signs and electrophysiological parameters^20^. The intramuscular fat fraction of the lower extremity, obtained using quantitative analyses of MRI fat fraction maps, reportedly correlates with CMTNSv2 in patients with CMT1A^23^. Quantitative analyses require the acquisition of an imaging sequence containing a fat fraction map that enables a direct quantitative measurement of the fat percentage of each muscle, which is not possible with conventional imaging sequences. Our proposed parameters obtained by a simple calculation of semiquantitative analyses result of muscles on conventional T1-weighted imaging sequence were also significantly correlated with CMTNSv2, which may imply a potential applicability of these parameters as imaging biomarkers. Studies with a larger number of participants are warranted to confirm our results.

We found positive correlations between MRI parameters measured for entire muscles and muscles at multiple levels and disease duration; nevertheless, these parameters were not significantly correlated with age at onset. These results may reflect the progressive nature of intramuscular fat infiltration in patients with CMT1A^2,3^. Earlier age at onset is reportedly related to a more severe disease course^18^, although we did not demonstrate any significant negative correlation between age at onset and MRI parameters.

The median Mercuri stage of the evaluated muscles was highest in the extensor hallucis longus and peroneus longus at level 5 (stage 2b). Other anterior and superficial posterior compartment muscles at level 5, as well as the peroneus longus and the gastrocnemius medialis at level 4, demonstrated a median stage of 2a. Price et al. divided the denervated muscles in the lower legs into ‘‘peroneal-type” and ‘‘tibial-type” muscles, based on the predominantly affected muscles^24^. Generally, patients with CMT1A present a peroneal-type pattern of fat infiltration, although superficial posterior compartment muscles are also commonly affected^25,26^. Our findings corroborate with previous study findings. Among the thigh muscles, the highest median Mercuri stage was found in the semitendinosus muscle at level 2 (stage 2a). Little has been reported on the pattern of thigh muscle denervation in patients with CMT1A. Hence, it would be interesting to investigate the tendency of muscle involvement based on disease severity in the future.

In addition to intrinsic limitations of retrospective studies, we acknowledge several limitations in our study. First, interobserver agreement was not evaluated due to the consensus-based analyses performed in this study. Second, imaging analyses were performed on non-dominant lower extremities. It may be more desirable to obtain imaging parameters that reflect comprehensive information on both lower extremity muscles. Third, the median FDS score of our cohort was 2 [1–2.5], indicating that the majority of patients could walk unaided. Thus, future studies should investigate whether our proposed MRI parameters are significantly correlated with clinical parameters in patients with more advanced disease stages. Fourth, it is not clear whether performing MRI analyses for individual muscles has clinical benefit over performing MRI analyses for individual muscle groups. Fifth, although our study results showed significant correlations between MRI parameters and clinical parameters, only mild to moderate correlations were demonstrated. It would be interesting to assess the relationships of MRI parameters with other clinical parameters such as those acquired using CMT pediatric scale, CMT functional outcome measure, 6-min walk test, or dynamometry. Sixth, the age of onset which was determined by asking the patients may not have been accurate due to inaccurate recall of initial symptom. In addition, we intended to reflect the severity of fat infiltration involving entire muscles at a designated level, and the degree of contribution of individual muscle compartments to the clinical severity could not be determined. This could be an interesting subject for investigation in future studies. Moreover, it would be beneficial to examine these patients in the long term, to assess whether these MRI parameters have potential applications in prognosis estimation.

In conclusion, we proposed preliminary MRI parameters that may comprehensively reflect the severity of intramuscular fat infiltration at designated levels of the lower extremity; these parameters were obtained through semiquantitative analyses of muscles based on the Mercuri scale in patients with CMT1A. These parameters were positively correlated with clinical parameters, thereby indicating their potential applicability as imaging biomarkers that clinically reflect disease severity in these patients. Regarding the degree and pattern of intramuscular fat infiltration, median Mercuri stage assessment showed a preferential involvement of the anterior and lateral compartment muscles in the distal lower leg.

## Supplementary Information

Supplementary Information.

## Data Availability

The datasets generated during and/or analyzed during the current study are available from the corresponding author on reasonable request.

## Abbreviations

CMT: Charcot-Marie-Tooth disease
PMP: Peripheral myelin protein
FIP: Fat infiltration proportion
SigFIP: Significant fat infiltration proportion
SevFIP: Severe fat infiltration proportion
CMTNSv2: Charcot-Marie-Tooth Neuropathy Score version 2
10-MWT: 10-M walk test
FDS: Functional disability scale
