# Supplementary material for: Magnetic resonance imaging-based lower limb muscle evaluation in Charcot-Marie-Tooth disease type 1A patients and its correlation with clinical data
Source: Sci Rep. 2022 Oct 5;12:16622. doi: 10.1038/s41598-022-21112-8 (PMC9534835; doi:10.1038/s41598-022-21112-8)
Supplement: Supplementary file 1 — Supplementary Information. [file 41598_2022_21112_MOESM1_ESM.docx]

**Supplementary Table S1.** Median Mercuri stage of evaluated thigh muscles in patients with Charcot-Marie-Tooth disease type 1A.

| **Level** | **Anterior compartment** | | | | | **Medial compartment** | | | | **Posterior compartment** | | |
| --- | --- | --- | --- | --- | --- | --- | --- | --- | --- | --- | --- | --- |
|  | **Sartorius** | **Rectus femoris** | **Vastus intermedius** | **Vastus lateralis** | **Vastus medialis** | **Adductor longus** | **Adductor brevis** | **Adductor magnus** | **Gracilis** | **Semitendinosus** | **Semimembranosus** | **Biceps femoris** |
| **Level 1** | 1 | 0 | 0 | 0 | 0 | 0 | 0 | 1 | 1 | 1 | NA | NA |
| **Level 2** | 1 | 0 | 0 | 0 | 0 | 0 | 0 | 1 | 1 | 2a | 1 | 1 |
| **Level 3** | 1 | 0 | 0 | 0 | 0 | NA | NA | NA | 1 | 1 | 1 | 1 |

Grading of intramuscular fat inﬁltration was performed using the Mercuri scale as follows: stage 0, normal appearance; stage 1, scattered small areas of increased signal; stage 2a, numerous discrete areas of increased signal comprising less than 30% of the muscle; stage 2b, numerous discrete areas of increased signal comprising 30–60% of the muscle; stage 3, washed-out appearance due to confluent areas of increased intensity with muscle still present at the periphery; stage 4, end-stage appearance with muscle entirely replaced by areas of increased signal.

NA, Not applicable.

**Supplementary Table S2.**  Median Mercuri stage of the evaluated leg muscles in patients with Charcot-Marie-Tooth disease type 1A.

| **Level** | **Anterior compartment** | | | **Lateral compartment** | **Superficial posterior compartment** | | | **Deep posterior compartment** | | |
| --- | --- | --- | --- | --- | --- | --- | --- | --- | --- | --- |
|  | **Tibialis anterior** | **Extensor digitorum longus** | **Extensor hallucis longus** | **Peroneus longus** | **Gastrocnemius medialis** | **Gastrocnemius lateralis** | **Soleus** | **Tibialis posterior** | **Flexor digitorum longus** | **Flexor hallucis longus** |
| **Level 4** | 1 | 1 | NA | 2a | 2a | 1 | 1 | 1 | NA | NA |
| **Level 5** | 2a | 2a | 2b | 2b | NA | NA | 2a | 1 | 1 | 1 |

Grading of intramuscular fat inﬁltration was performed using the Mercuri scale as follows: stage 0, normal appearance; stage 1, scattered small areas of increased signal; stage 2a, numerous discrete areas of increased signal comprising less than 30% of the muscle; stage 2b, numerous discrete areas of increased signal comprising 30–60% of the muscle; stage 3, washed-out appearance due to confluent areas of increased intensity with muscle still present at the periphery; stage 4 end-stage appearance with muscle entirely replaced by areas of increased signal.

NA, Not applicable.

**Supplementary Table S3.** MRI analysis results in patients with Charcot-Marie-Tooth disease type 1A.

| **Level** | **FIP** | **SigFIP** | **SevFIP** |
| --- | --- | --- | --- |
| **Total** | 60.9±22.7% (45.7-73.9%) | 23.9±23.2% (8.7-43.5%) | 2.2±11.4% (0.0-14.7%) |
| **Level 1** | 40.0±26.2% (20.0-40.0%) | 0.0±17.6% (0.0-10.0%) | 0.0±0.8% (0.0-0.0%) |
| **Level 2** | 50.0±22.9% (41.7-66.7%) | 8.3±22.3% (0.0-39.6%) | 0.0±4.2% (0.0-0.0%) |
| **Level 3** | 55.6±24.7% (55.6-88.9%) | 0.0±29.3% (0.0-55.6%) | 0.0±6.1% (0.0-0.0%) |
| **Level 4** | 92.9±38.0% (42.9-100.0%) | 42.9±39.3% (0.0-85.7%) | 0.0±29.4% (0.0-28.6%) |
| **Level 5** | 100.0±35.5% (62.5-100.0%) | 62.5±41.0% (12.5-100.0%) | 12.5±37.8% (0.0-50.0%) |

Data are presented as median ± standard deviation, with interquartile ranges in parentheses.

FIP, fat infiltration proportion; SigFIP, significant fat infiltration proportion; SevFIP, severe fat infiltration proportion
